# Reactive Transformation and Increased BDNF Signaling by Hippocampal Astrocytes in Response to MK-801

**DOI:** 10.1371/journal.pone.0145651

**Published:** 2015-12-23

**Authors:** Wenjuan Yu, Hao Zhu, Yueming Wang, Guanjun Li, Lihua Wang, Huafang Li

**Affiliations:** 1 Shanghai Mental Health Center, Shanghai Jiao Tong University School of Medicine, Shanghai, China; 2 Department of Anesthesiology, Renji Hospital, Shanghai Jiao Tong University School of Medicine, Shanghai, China; 3 Department of Anatomy, Shanghai Jiao Tong University School of Medicine, Shanghai, China; 4 Shanghai Key Laboratory of Psychotic Disorders, Shanghai, China; Albany Medical College, UNITED STATES

## Abstract

MK-801, also known as dizocilpine, is a noncompetitive N-methyl-D-aspartic acid (NMDA) receptor antagonist that induces schizophrenia-like symptoms. While astrocytes have been implicated in the pathophysiology of psychiatric disorders, including schizophrenia, astrocytic responses to MK-801 and their significance to schizotypic symptoms are unclear. Changes in the expression levels of glial fibrillary acid protein (GFAP), a marker of astrocyte activation in response to a variety of pathogenic stimuli, were examined in the hippocampus of rats treated with the repeated MK-801 injection (0.5 mg/10ml/kg body weight for 6 days) and in primary cultured hippocampal astrocytes incubated with MK-801 (5 or 20 μM for 24 h). Moreover, the expression levels of BDNF and its receptors TrkB and p75 were examined in MK-801-treated astrocyte cultures. MK-801 treatment enhanced GFAP expression in the rat hippocampus and also increased the levels of GFAP protein and mRNA in hippocampal astrocytes *in vitro*. Treatment of cultured hippocampal astrocytes with MK-801 enhanced protein and mRNA levels of BDNF, TrkB, and p75. Collectively, our results suggest that hippocampal astrocytes may contribute to the pathophysiology of schizophrenia symptoms associated with NMDA receptor hypofunction by reactive transformation and altered BDNF signaling.

## Introduction

Schizophrenia is a chronic and debilitating syndrome that afflicts approximately 1% of the population worldwide [1]. The N-methyl-D-aspartic acid (NMDA) receptor hypofunction hypothesis, originally proposed by Olney and Farber [2], suggests that dysfunctional glutamatergic neurotransmission contributes to the pathophysiology of schizophrenia. MK-801 (dizocilpine) is a noncompetitive N-methyl-D-aspartic acid (NMDA) receptor antagonist with a favorable profile compared to other NMDA receptor antagonists because it has an extremely high affinity (10−100 fold higher than PCP and ketamine) [3] and selectivity for the receptor PCP binding site [4]. MK-801 is known to induce schizophrenia-like symptoms [2]. Our previous study indicated that repeated high doses (0.5 mg/kg every day for six days) of MK-801 in rats induced schizophrenia-like behaviors [5].

Astrocytes, the most abundant type of glial cell in the central nervous system, are crucial for neuroplasticity and neural homeostasis across life-span. Astrocytes are now emerging as key participants in many aspects of brain disease. Astrocytes regulate neuronal excitability by transporting extracellular glutamate, adenosine, potassium, lactate, and GABA among other neurotransmitters and neuromodulators, while disruption of these transport functions has been implicated in the pathogenesis of epilepsy [6]. Astrocytes are also implicated in the pathophysiology of psychiatric illnesses, including schizophrenia [7,8].

Astrocytes synthesize and secrete a large number of cytokines that regulate neural plasticity and response to injury, including brain-derived neurotrophic factor (BDNF) [9]. BDNF is a member of the neurotrophin family, proteins originally identified as neuronal survival factors [10]. In addition to promoting proliferation and differentiation of neurons, BDNF influences the shape and number of dendritic spines, a critical determinant of neural information processing capacity [11,12]. Furthermore, BDNF regulates forms of hippocampal synaptic plasticity linked to learning and memory, and age-related decline in BDNF signaling may contribute to age-related memory deficits [12,13]. By interacting with p75NTR and TrkB receptors, BDNF facilitate long-term depression (LTD) and long-term potentiation (LTP), respectively, two forms of synaptic plasticity implicated in hippocampus-dependent learning [14]. Deficits in these synaptoplastic processes could underlie some of the cognitive deficits exhibited by schizophrenia patients. Moreover, BDNF is released by astrocytes in response to physiological and pathological signals from neurons, a form of neuron-glial signaling that may regulate neuroplasticity and enhance neuronal resistance to injury [15,16].

A previous study indicated that BDNF expression in the hippocampus is significantly upregulated in MK-801-treated rats [17]. However, the effects of MK-801 on hippocampal astrocytes poorly investigated. Therefore, in the current study, we evaluated GFAP, BDNF, TrkB, and p75 expression levels in hippocampal astrocytes in the animal model of schizophrenia based on the repeated treatment with MK-801 and/or in primary cultures of hippocampal astrocytes.

## Experimental Procedures

### 2.1. Rats and drug treatment

Sixteen male Sprague-Dawley rats (210–240 g) from the Animal Care Facility at Shanghai Jiao Tong University School of Medicine were used for these experiments. Rats were housed in pairs on a 12 h light/dark cycle and given one week to acclimate to the housing conditions with food and water ad libitum prior to MK-801 treatment. Dizocilpine (MK-801; [5R, 10S]-[+]-5-methyl- 10,11-dihydro-5H-dibenzo[a,d] cyclohepten-5,10-imine; Sigma-Aldrich, St Louis, MO, USA) was dissolved in saline and administered by intraperitoneal injection (n = 8 rats; 0.5 mg/10ml/kg body weight) daily for 6 days. Control animals (n = 8) received equal volumes of normal saline. All experimental procedures were in accordance with the National Institutes of Health Guide for the Care and Use of Laboratory Animals (Publication No. 80–23, revised in 1996) and approved by the Animal Care Committee of the Laboratory Animal at Shanghai Jiao Tong University School of Medicine.

### 2.2. Immunohistohemistry

One hour after the last MK-801 injection, rats (n = 4 per group) were deeply anaesthetized with sodium pentobarbital (100 mg/kg i.p.) and transcardially perfused with 100 ml saline solution followed by 400 ml of 4% paraformaldehyde in 0.01M phosphate-buffered saline (PBS, pH 7.4). Brains were immediately removed, post-fixed for 2 h in the same fixative, and cryoprotected in 20% sucrose solution at 4°C for 24 h. Serial coronal sections of 15-μm thickness at various levels (75 μm interval) were cut through the hippocampus on a freezing microtome (Leitz, Wetzlar, Germany) and slide-mounted. Six sections were selected from the right or left hippocampus respectively per each rat. The sections were incubated in 3% hydrogen peroxide to quench endogenous peroxidase activity, blocked and permeabilized in 0.01M PBS with 1% bovine serum albumin (BSA; Gibco) and 0.3% Triton X-100 for 1 h at room temperature (RT), and then incubated with anti-GFAP (1:500, Sigma) overnight at 4°C. The next morning, sections were incubated with a horseradish peroxidase (HRP)-conjugated secondary antibody (Sigma) for 2 h at RT. Finally, immunostaining was visualized by brief incubation in a solution of 0.05% 3,3-diaminobenzidine (DAB) and 3% hydrogen peroxide in 0.01M PBS. Specimens were dehydrated, mounted, and photographed using a Leica DM6000 microscope with a CCD 2/3 camera. We selected four random squares (300 μm ×300 μm) from each section. Images were converted to grey scale designated as optical density (OD) and analyzed with RS IMAGE ProTM Version4.5 (Roper Scientific, Trenton, NJ, USA).

### 2.3. Primary astrocyte cultures

Astrocyte cultures were established using our previous method [18]. Briefly, astrocyte cultures were prepared from the hippocampi of 2-day-old neonatal Sprague–Dawley rats following mechanical dissociation. Dissociated cells were suspended in Dulbecco's Modified Eagle's Medium (DMEM) (Gibco, Invitrogen, Grand Island, NY) supplemented with 10% fetal bovine serum (FBS; Gibco) and 1 mM glutamine (Gibco). Cells were then seeded on uncoated 25-cm^2^ flask at 200,000 cells/cm^2^. Medium was changed 2 days after initiation of culture and twice per week thereafter. When cultures reached confluence (10 to 11 days after plating), non-astrocytes, such as microglia, were detached from the flasks by shaking and the medium was replaced. Astrocytes were detached using 0.25% EDTA-trypsin (Sigma, St. Louis, MO, USA) and passaged. Experiments were initiated after the second passage.

### 2.4. Western blotting

One hour after the last MK-801 injection, rats (n = 4 per group) were deeply anaesthetized with sodium pentobarbital (100 mg/kg i.p.) and transcardially perfused with 100 ml saline solution. Hippocampi were immediately removed. Cultured astrocytes and hippocampi were lysed in buffer (50 mM Tris–HCl, pH 7.5, 150 mM NaCl, 1% NP-40, 0.5% sodium deoxycholate, 0.1% SDS, 1 mM EDTA, 1 mM sodium orthovanadate, 10 mM sodium fluoride, 4 μg/mL leupeptin, 1 μg/mL aprotinin, and 100 μg/mL PMSF; all from Sigma). After incubation on ice for 15 min, homogenates were clarified by centrifugation at 12,000×g for 10 min at 4°C and the supernatant collected. The protein concentration was determined by BCA assay. For Western blotting, 20 μg cellular protein per gel lane was electrophoresed on 12% SDS/polyacrylamide gels and transferred to nitrocellulose membranes. after blocking in 5% non-fat dry milk dissolved in Tris-buffered saline plus 0.1% Tween 20 (TBS-T) and washing with TBS-T, membranes were incubated overnight with anti-GFAP (1:2000, Sigma), anti-BDNF (1:400, Santa Cruz Biotechnology, Santa Cruz, CA), anti-TrkB (1:2000, Sigma), anti-p75 (1:2000, Sigma), and anti-GAPDH (1:5000, Sigma). The next day, the membranes were incubated with an HRP-conjugated secondary antibody (1:1000, Sigma) for 1 h following washes with TBS-T. Immunolabeling was detected by an enhanced chemiluminescence (ECL) detection kit (Pierce, Rockford, IL, USA).

### 2.5. Real-time reverse transcription-polymerase chain reaction

Real-time PCR were processed according to our previous protocols [19]. Briefly, astrocytes were incubated in serum-free DMEM containing 0, 5 or 20 μM MK-801 for 24 h and washed with PBS. Total RNA was extracted using TRIzol reagent (Invitrogen, USA) and reverse transcribed using the PrimeScriptTM RT Reagent Kit (Perfect Real Time) (TaKaRa Biotechnology, Japan). Expression levels of GFAP, BDNF, TrkB, p75, and GAPDH (control) mRNA were quantified by a Roche Light Cycler system using the QuantiTect SYBR Green PCR kit (QuantiTect, Qiagen, Valencia, CA). The sequences of forward and reverse primers are listed in Table 1. Expression of each gene was normalized to the mean Ct value of housekeeping gene GAPDH in the PCR array. Differences in expression between treatment groups were calculated by the ΔΔCt method and the values are expressed as 2^-ΔΔCt^. Each trial was performed in triplicate.

**Table 1:** The sequences of gene-specific primers used for qRT-PCR.

Gene name	Forward (5'-3')	Reverse (5'-3')
GFAP	TGGCCACCAGTAACATGCAA	CAGTTGGCGGCGATAGTCAT
BDNF	GTCACAGCGGCAGATAAAAAG	ATGGGATTACACTTGGTCTCGT
TrkB	CGACACTCAGGATTTGTATTGC	ATGGTCACAGACTTCCCTTCC
p75	AGCAGACCCATACGCAGACT	GCAGTTTCTCTACCTCCTCAC
GAPDH	AGGGTGGTGGACCTCATGG	AGCAACTGAGGGCCTCTCTCTT

### 2.6. Statistical analysis

All results were expressed as mean ± SEM. A two-tailed t test for independent samples was used for two-group comparisons. One-way ANOVA followed by Newman-Keuls multiple comparison tests were used to compare results from control and MK801-treated groups. A *P*<0.05 was considered statistically significant.

## Results

### 3.1. MK-801 activated hippocampal astrocytes in vivo

**Fig 1:**
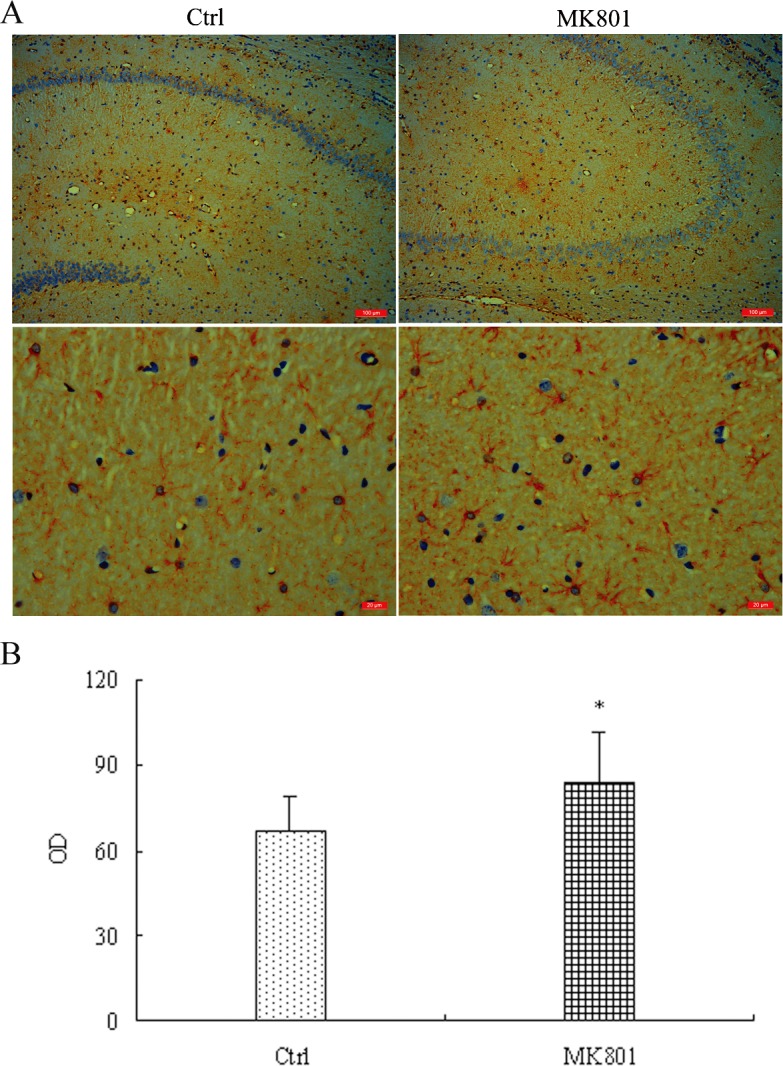
Enhanced GFAP immunoreactivity in hippocampal astrocytes of MK-801-treated rats. MK-801 was administered by intraperitoneal injection (0.5 mg/10ml/kg body weight) daily for 6 days. Control (Ctrl) animals received equal volumes of normal saline. (A) Extensive positive cellular labeling of GFAP was seen in MK801-induced rat hippocampus (n = 4 rats per group) while only mild positive staining was observed in control group. (B) Computer assisted image analysis revealed significantly increased GFAP expression in MK-801-treated rats compared to saline-treated rats. OD = optical density. Data are the mean±S.E.M. Statistical differences between two groups were determined by student's t-tests. *P <0.05 vs. control group.

**Fig 2:**
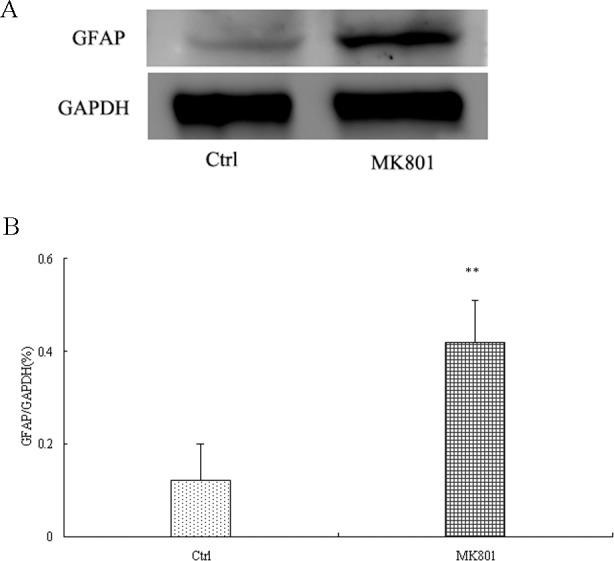
Enhanced GFAP protein expression in the hippocampus of MK-801-treated rats as measured by western blotting. MK-801 was administered by intraperitoneal injection (0.5 mg/10ml/kg body weight) daily for 6 days. Control animals received equal volumes of normal saline. GFAP expression was detected by western blotting of hippocampal lysates from MK-801-treated (MK801) and Control (Ctrl) rats (n = 4 per group). Densitometric values expressed as mean ± S.E.M relative to the gel loading control. Statistical differences between two groups were determined by student's t-tests. **P<0.01 vs. control group.

**Fig 3:**
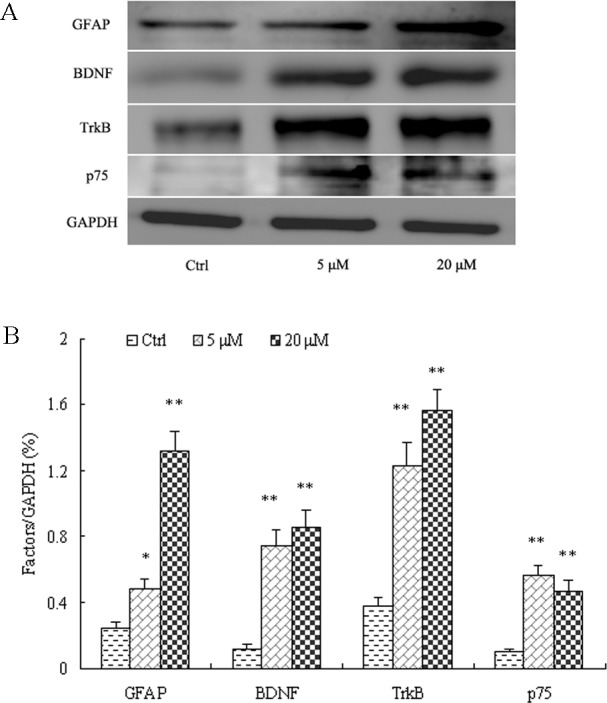
MK-801 enhanced protein expressions levels of GFAP, BDNF, TrkB, and p75 in culture hippocampal astrocytes. Densitometric values are expressed as the mean ± S.E.M of 3 independent experiments. Statistical differences between groups were determined by one-way ANOVA followed by Newman-Keuls multiple comparison tests. *P<0.05 and **P<0.01 vs. control group.

Glial fibrillary acidic protein (GFAP), an intermediate filament (IF) protein, is expressed in the central nervous system in astrocyte cells and used as a marker of astrocyte activation. Consistent with astroglial activation by MK-801, GFAP immunostaining was markedly more intense in the MK-801-treated rat group than saline-treated controls as revealed by computer-assisted image analysis (67 ± 12 vs. 84 ± 18; *t*_6_ = 2.752, *P*<0.05) (Fig 1). This increase in GFAP expression was confirmed by Western blotting of lysate from hippocampus; GFAP expression was enhanced about 4-fold in MK-801-treated hippocampus relative to control (*P*<0.01) (Fig 2). Additionally, GFAP expression was higher in cultured hippocampal astrocytes after a 24-h treatment with 5 μM MK-801 (*P*<0.05) compared to control cultures and elevated even further after 20 μM MK-801 treatment (*P*<0.01) (Fig 3).

### 3.2. MK-801 upregulated the protein expression of BDNF and its receptors in vitro

Astrocytes synthesize and secrete a variety of neurotrophic factors including BDNF. Astrocytes also express BDNF receptors p75 and TrkB, suggesting both autocrine and neuron-to-glial BDNF signaling. Moreover, aberrant regulation of BDNF and its receptors may be involved in the pathophysiology of schizophrenia. In cultured hippocampal astrocytes, MK-801 (5 and 20 μM for 24 h) increased BDNF protein expression relative to untreated controls (6.2-fold at 5 μM and 7.2-fold increase at 20 μM MK801; *P*<0.01 for both) (Fig 3). Treatment with MK-801 also increased the expression of TrkB (3.2-fold at 5 μM, 4.2-fold at 20 μM) and p75 (5.6-fold at 5 μM, 4.7-fold at 20 μM) (Fig 3).

### 3.3. MK-801 enhanced mRNA levels of GFAP, BDNF, TrkB, and p75

**Fig 4:**
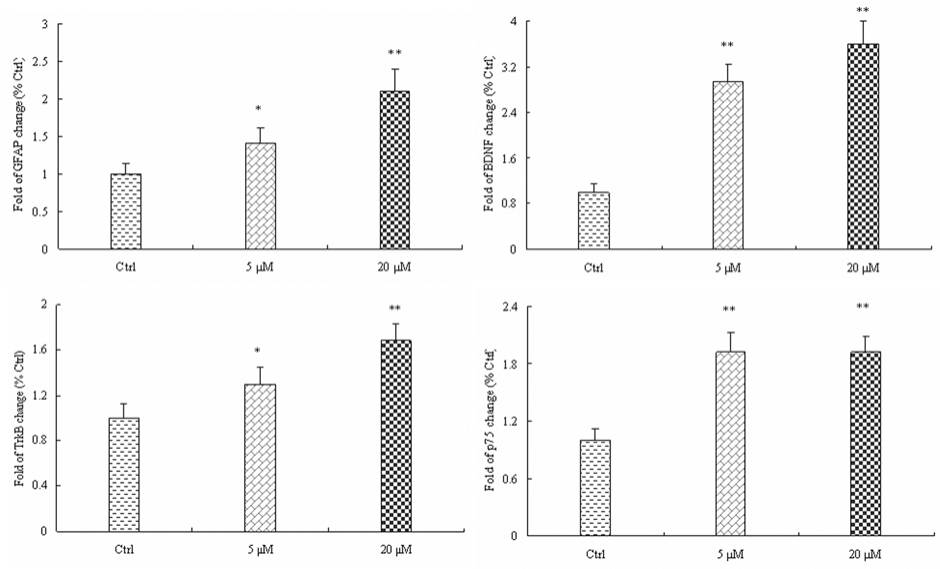
The mRNA expression levels of GFAP, BDNF, TrkB, and p75 were enhanced by MK-801 in cultured hippocampal astrocytes. Densitometric values are expressed as the mean ± S.E.M of 3 independent experiments. Statistical differences between groups were determined by one-way ANOVA followed by Newman-Keuls multiple comparison tests. *P<0.05 and **P<0.01 vs. control group.

The mRNA expression levels of GFAP, BDNF, TrkB, and p75 in cultured hippocampal astrocytes were measured by real-time PCR following treatment with 5 or 20 μM MK-801 for 24 h. Consistent with Western blotting results, GFAP mRNA was increased 1.4-fold versus control at 5 μM MK-801 (*P*<0.05) and 2.1-fold at 20 μM MK-801 (*P*<0.01), while expression of BDNF mRNA increased by 2.9- and 3.58-fold, respectively (*P*<0.01 for both) (Fig 4). In addition, TrkB mRNA expression was increased by 1.3-fold at 5 μM (*P*<0.05) and 1.7-fold at 20 μM MK801 (*P*<0.01). The expression of p75 mRNA was upregulated by 1.9-fold at both 5 and 20 μM MK801 (*P*<0.01 for both) (Fig 4).

## Discussion

In the present study, the hippocampal astrocytes were activated by MK-801 treatment in vivo and in vitro. MK-801 is a noncompetitive NMDA receptor antagonist shown to cause strong psychomimetic effects such as hallucinations and psychomotor signs, and thus has been used extensively in schizophrenia research. Our previous study indicated that rats administered repeated high doses (0.5 mg/kg every day for six days) of MK-801 exhibited schizophrenia-like behaviors [5]. Kondziella et al. [20] reported that a higher repeated dose (0.5 mg/kg) decreased labeling of glutamate and GABA from [1-^13^C] glucose in the FCR, mimicking the alterations observed in patients with schizophrenia [21,22]. Repeated high doses (0.5 mg/kg) also mimic some of the behavioral characteristics, such as mild hyperlocomotion and prepulse inhibition disruption, and the neurochemical alterations seen in other animal model of schizophrenia [23].

In this study, repeated high doses of MK-801 significantly increased the expressions of GFAP in the rat hippocampus. GFAP is an intermediate filament protein expressed specifically by astrocytes in the central nervous system. Enhanced expression is a marker of astrocyte activation, a cluster of reactive morphological and physiological changes in response to acute and chronic brain injury. Hippocampal astrocytes were activated by MK-801 in vivo, consistent with a previous study [24]. Repeated intraperitoneal injections of another NMDA receptor antagonist, phencyclidine (PCP), for 7 days also enhanced GFAP expression in the hippocampus [25]. However, Gomes et al. reported no change in GFAP expression in the dorsal hippocampus of mice treated with MK-801 for 28 days [26]. Thus, the astrocytic response to MK-801 is highly dose- and time-dependent in rats.

To examine the direct effects of MK-801 on hippocampal astrocytes, primary astrocyte cultures were studied. Consistent with the results in vivo, MK-801 enhanced GFAP expression at both the protein and mRNA levels. Evidence indicates that chronic MK-801 treatment of cultured astrocytes (>36 h) causes substantial cytotoxicity [27,28]. However, we found no significant apoptosis of hippocampal astrocytes in response to 20 μM MK-801 as measured by flow cytometry (data not shown). This discrepancy may stem from differences in treatment times and the specific astrocyte lineage. In our study, primary rat hippocampal astrocyte cultures were used, while a human astrocytoma cell line, 1321 N1, has been selected for other [27,28].

Activation of astrocytes alters the expression levels of molecules involved in metabolism, neurotransmitters release, neuroplasticity, and neuron-glial signaling. Reactive astrocytes produce neurotrophic factors, including BDNF [29]. Astrocytes also express the BDNF receptors, TrkB and p75, and schizophrenia is closely related to imbalanced circuit-level expression of BDNF signaling molecules [30]. BDNF signaling is thought to play an important role in the pathophysiology of schizophrenia, but the contribution of BDNF signaling by hippocampal astrocytes is currently unclear. In this study, MK-801 significantly increased the expression levels of BDNF, TrkB, and p75 in hippocampal astrocytes in vitro. MK-801 binds inside the ion channel of the receptor at several PCP binding sites, thereby preventing the flow of ions through the channel, including Ca2+ [31]. In astrocytes, however, glutamate- and NMDA-evoked [Ca2+]i elevations were not blocked by MK-801 [31,32], suggesting that astrocytic NMDARs function in a non-canonical, Ca2+ flux-independent manner. Alternatively, AMPA/kainate receptors and mGluRs may be critical for glutamate-evoked [Ca2+]i increases in astrocytes [31]. Thus, MK-801-evoked BDNF upregulation and signaling may not be related to NMDAR-dependent calcium influx in astrocytes.

The MEK−MAPK and PI3K−Akt−GSK-3β pathways are the primary signaling cascades exploited by NMDA receptors. Seo et al. reported that repeated treatment with 1 mg/kg MK-801 enhanced the phosphorylation levels of several signaling molecules in rat brain, including the Akt−GSK3β and MEK−ERK pathways and the transcription factor CREB [33]. And another study found that after single injection of 1 mg/kg MK-801, the phosphorylation of Ser9-GSK-3β was increased from 15 min compared to the control, and the phosphorylation of both Ser473-Akt and Ser133-CREB, upstream and downstream molecules of GSK-3β respectively, followed the same temporal course after single injection of 1 mg/kg MK-801 [34]. These signaling factors are not only associated with the pathophysiology of schizophrenia, but are also involved in the regulation of BDNF signaling. Moreover, MK-801-induced upregulation of BDNF signaling was blocked by PD98059, an inhibitor of ERK pathways, and by LY29400, an inhibitor of PI3K pathways (data not shown), so MK-801 may regulate BDNF signaling through ERK and PI3K−Akt pathways.

BDNF is synthesized and secreted into the intercellular space. Transcriptional upregulation results from TrkB-mediated phosphorylation of CREB, which then binds to cAMP response element sites in the BDNF promoter to elevate gene transcription [35]. In astrocytes, BDNF binding to TrkB activates a Rho GTPase to promote glycine transporter (GlyT) internalization through a dynamin/clathrin-dependent mechanism, thereby reducing glycine uptake [36]. Moreover, BDNF also influences GAT-1 mediated GABA transport, and increases GABA uptake into astrocytes [37]. In addition, BDNF has been shown to prevent phencyclidine-induced apoptosis in developing brain [38] and MK-801-induced apoptotic neuronal death in immature neuronal cultures [39]. BDNF derived from hippocampal astrocytes can act through neurogenesis-dependent and independent mechanisms to regulate different facets of anxiolytic-like responses [40].

In the healthy adult rat CNS, the expression of p75 is restricted to a small population of astrocytes, but expression is strongly upregulated by injury [41]. Induced p75 not only activates downstream signaling cascades upon BDNF binding to promote neurite growth, but also increases the neurotrophin binding affinity to Trk and promotes Trk signaling [42]. Specifically, p75 can alter a subdomain of the Trk receptor to expose a special site for neurotrophin binding, enhancing binding affinity (K_d_) of the Trk receptor from 10^−9^ M to 10^−11^ M [43]. BDNF, by interacting with p75 and TrkB receptors, facilitates both LTD and LTP [14].

Altered BDNF signaling has been demonstrated in several regions of the schizophrenia brain by post mortem studies. However, there is conflicting evidence regarding changes in BDNF signaling in the hippocampus of patients with schizophrenia. Several studies have detected enhanced BDNF expression, while others have reported decreased BDNF and TrkB receptor expression [30]. It is possible that disease stage or antipsychotic treatment history may influence BDNF signaling. Similarly, there are consistencies regarding changes in BDNF signaling in the animal model of schizophrenia based on antagonism of NMDA receptors, as both decreased BDNF protein levels in the hippocampus [44] and elevated BDNF [17] have been reported. These inconsistencies may reflect differences in BDNF responses dependent on animal age, drug treatment duration, and/or brain region. For instance, perinatal phencyclidine treatment significantly reduced p75 expression in juvenile rats but increased expression in adult rats [45].

This study has several limitations. First, most results were obtained from cultured hippocampal astrocytes. Notably, changes in BDNF signaling were not examined in vivo, where the response could differ from that in culture due to compensatory changes induced by suppression of NMDA receptor signaling, such as alterations in GABAergic, serotonergic, and adrenergic neurotransmitter systems. Second, the role of hippocampal astroglial BDNF signaling in the pathophysiology of this schizophrenia model was not directly examined.

In conclusion, astrocytes react to injury or stress by increasing expression of several proteins implicated in schizophrenia, including BDNF and its receptors. Our findings suggest that hippocampal astrocytes may participate in the pathogenesis of schizophrenia symptoms associated with NMDA receptor hypofunction, specifically by reactive transformation and altered BDNF signaling. Additional studies are currently being performed at our institute to further elucidate the involvement of astroglial factors in the animal model of schizophrenia based on antagonism of NMDA receptors.

## Supporting Information

S1 Fig The mRNA levels of BDNF were detected through real-time PCR in hippocampal astrocytes. Hippocampal astrocytes were treated with 20 uM MK-801 or 10 uM ketamine for 24 h. And the hippocampal astrocytes were incubated with 2 uM NMDA in the absence and in the presence of 20 uM MK-801for 24 h. BDNF mRNA level was significantly elevated in the astrocytes incubated with MK-801 or ketamine, and reduced in astrocytes with NMDA treatment. However, reduced BDNF mRNA level was reversed in the presence of MK-801. These results suggested that MK-801 may regulate BDNF signaling through NMDA receptor. Values of densitometric analysis are the means ± S.E.M of 3 independent experiments. Statistical differences between groups were determined by student's t-tests. *P <0.05 and **P<0.01 vs. control group.
(TIF)

S2 Fig The cell apoptosis were detected in hippocampal astrocytes incubated with MK801 for 48h by flow cytometry (A and B). The cells were treated with 20 uM MK801 at 0 h in MK801(20uM/48h) group, and with 20 uM MK801 twice, at 0 h and 24 h in another group. The apoptosis was not induced in hippocampal astrocytes with 20 uM MK801 treatment for 48h. Even another 20 uM MK801 was added to proceed to incubate astrocytes after 24 h, the apoptosis did not also appear. Values of densitometric analysis are the means ± S.E.M of 3 independent experiments.
(TIF)

S3 Fig BDNF mRNA expression were detected in hippocampal astrocytes incubated with MK801 for 48h by real-time PCR. The cells were treated with 20 uM MK801 at 0 h in MK801(20uM/48h) group, and with 20 uM MK801 twice, at 0 h and 24 h in another group. BDNF mRNA level was elevated in hippocampal astrocytes treated with MK801 at 24 h, and subsequently declined to normalization at 48 h. However, BDNF mRNA level was enhanced by another 20 uM MK801 addition at 48 h. Values of densitometric analysis are the means ± S.E.M of 3 independent experiments. Statistical differences between groups were determined by student's t-tests. **P<0.01 vs. control group.
(TIF)

S4 Fig Western blot analysis of GFAP in rat hippocampus. (TIF)

S5 Fig Western blot analysis of GFAP in vitro. (TIF)

S6 Fig Western blot analysis of BDNF in vitro. (TIF)

S7 Fig Western blot analysis of TrkB in vitro. (TIF)

S8 Fig Western blot analysis of p75 in vitro. (TIF)

S9 Fig The data of RT-PCR. (TIF)

S1 Table The data of GFAP immunoreactivity. (DOCX)

S2 Table The data of GFAP protein by western blotting in vivo. (DOCX)

S3 Table The data of GFAP protein by western blotting in vitro. (DOCX)

S4 Table The data of BDNF protein by western blotting in vitro. (DOCX)

S5 Table The data of TrkB protein by western blotting in vitro. (DOCX)

S6 Table The data of p75 protein by western blotting in vitro. (DOCX)
