# Supplementary figures and images for: Reactive Transformation and Increased BDNF Signaling by Hippocampal Astrocytes in Response to MK-801
Source: PLoS One. 2015 Dec 23;10(12):e0145651. doi: 10.1371/journal.pone.0145651 (PMC4689377; doi:10.1371/journal.pone.0145651)

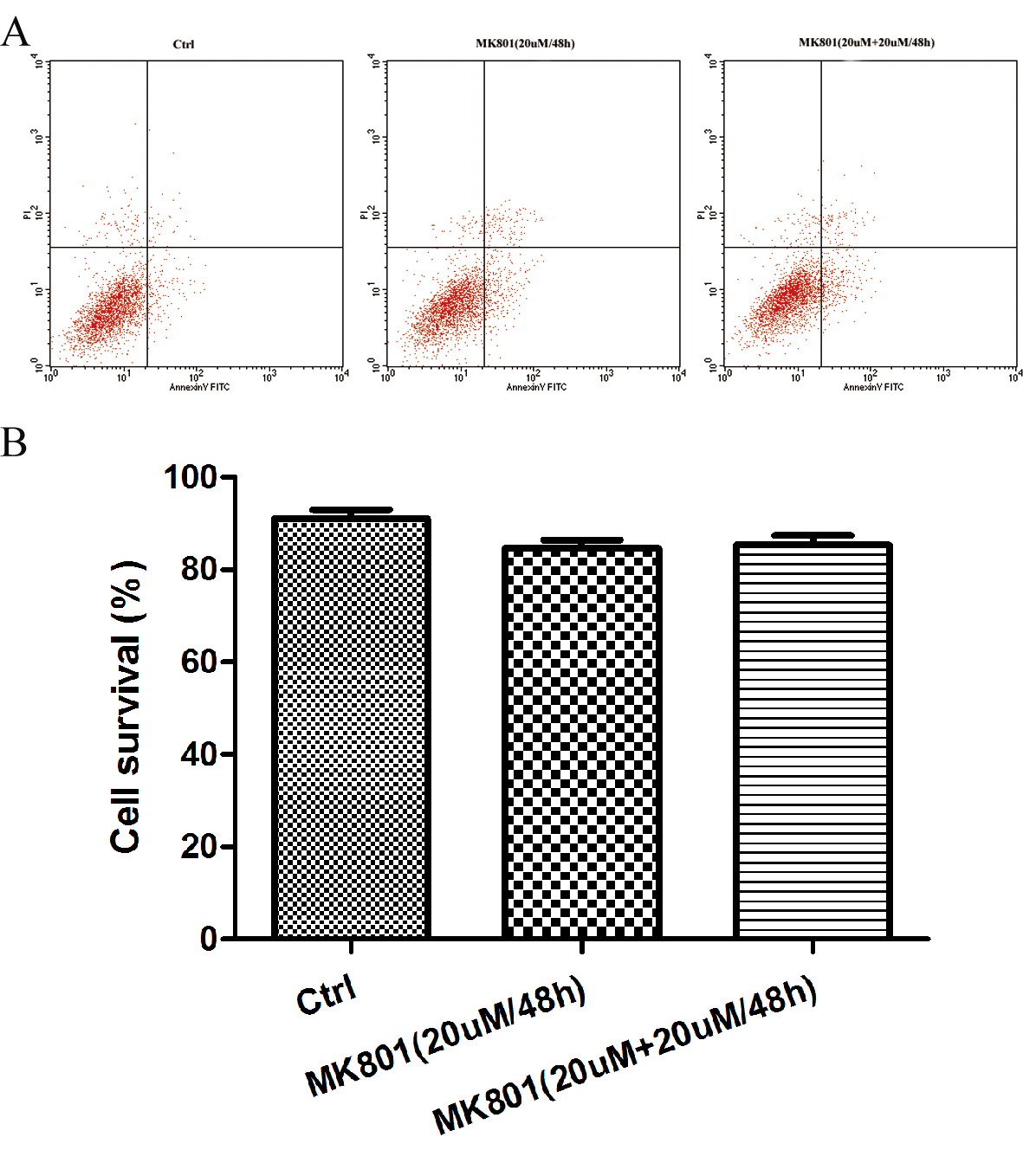

Supplement: S2 Fig — The cells were treated with 20 uM MK801 at 0 h in MK801(20uM/48h) group, and with 20 uM MK801 twice, at 0 h and 24 h in another group. The apoptosis was not induced in hippocampal astrocytes with 20 uM MK801 treatment for 48h. Even another 20 uM MK801 was added to proceed to incubate astrocytes after 24 h, the apoptosis did not also appear. Values of densitometric analysis are the means ± S.E.M of 3 independent experiments. (TIF) [file pone.0145651.s002.tif]

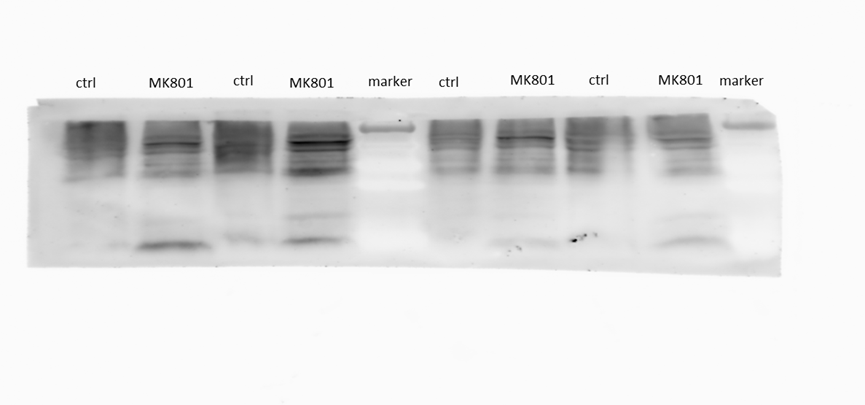

Supplement: S4 Fig — (TIF) [file pone.0145651.s004.tif]

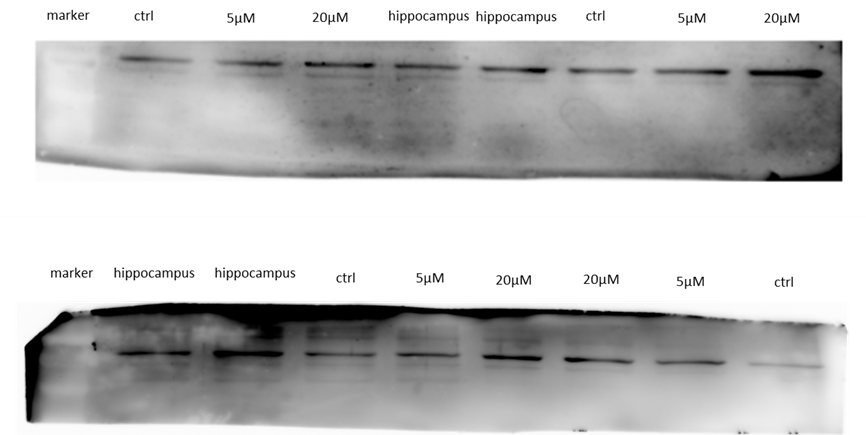

Supplement: S5 Fig — (TIF) [file pone.0145651.s005.tif]

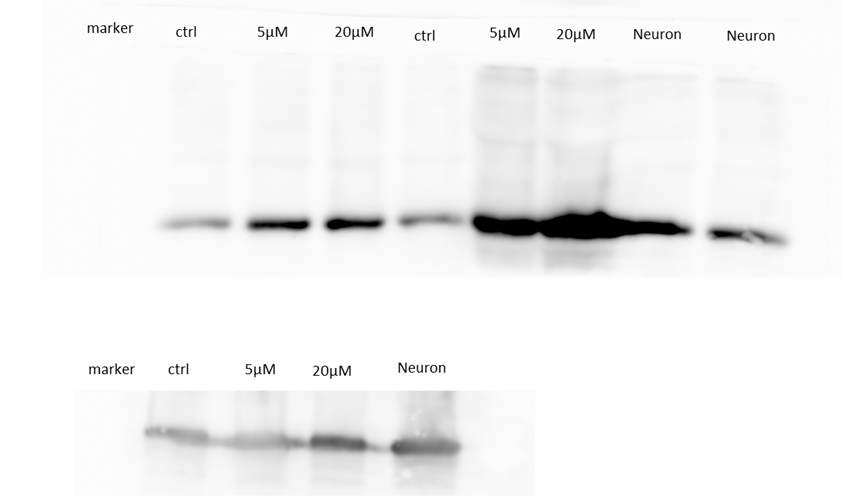

Supplement: S6 Fig — (TIF) [file pone.0145651.s006.tif]

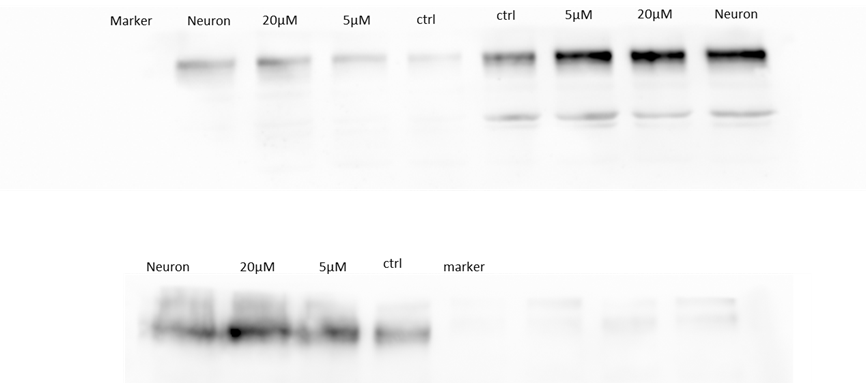

Supplement: S7 Fig — (TIF) [file pone.0145651.s007.tif]

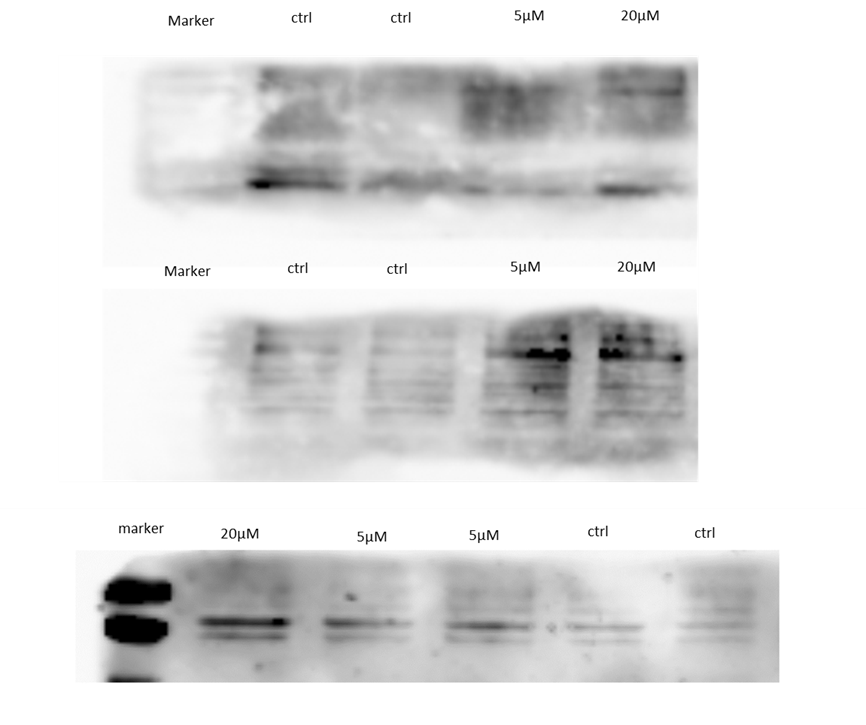

Supplement: S8 Fig — (TIF) [file pone.0145651.s008.tif]

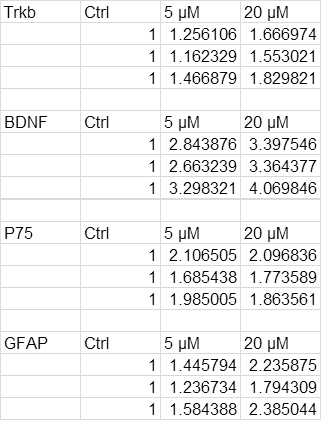

Supplement: S9 Fig — (TIF) [file pone.0145651.s009.tif]
