# Supplementary material for: Reactive Transformation and Increased BDNF Signaling by Hippocampal Astrocytes in Response to MK-801
Source: PLoS One. 2015 Dec 23;10(12):e0145651. doi: 10.1371/journal.pone.0145651 (PMC4689377; doi:10.1371/journal.pone.0145651)
Supplement: S1 Table — (DOCX) [file pone.0145651.s010.docx]

**S1 Table. The data of GFAP immunoreactivity**

| GFAP | OD | |
| --- | --- | --- |
|  | Ctrl | Mk801 |
|  | 69 | 98 |
|  | 76 | 82 |
|  | 66 | 79 |
|  | 54 | 78 |
