# Supplementary material for: Reactive Transformation and Increased BDNF Signaling by Hippocampal Astrocytes in Response to MK-801
Source: PLoS One. 2015 Dec 23;10(12):e0145651. doi: 10.1371/journal.pone.0145651 (PMC4689377; doi:10.1371/journal.pone.0145651)
Supplement: S2 Table — (DOCX) [file pone.0145651.s011.docx]

**S2 Table. The data of GFAP protein by western blotting in vivo**

| GFAP  (in vivo) | IOD | |
| --- | --- | --- |
|  | Ctrl | MK801 |
|  | 510 | 1601 |
|  | 764 | 2756 |
|  | 349 | 1446 |
|  | 288 | 1195 |
