# Supplementary material for: Reactive Transformation and Increased BDNF Signaling by Hippocampal Astrocytes in Response to MK-801
Source: PLoS One. 2015 Dec 23;10(12):e0145651. doi: 10.1371/journal.pone.0145651 (PMC4689377; doi:10.1371/journal.pone.0145651)
Supplement: S3 Table — (DOCX) [file pone.0145651.s012.docx]

**S3 Table. The data of GFAP protein by western blotting in vitro**

| GFAP | IOD | | |
| --- | --- | --- | --- |
|  | Ctrl | 5 uM | 20 uM |
|  | 1203 | 2453 | 3987 |
|  | 1754 | 2339 | 8959 |
|  | 1967 | 2649 | 5825 |
|  | 836 | 2253 | 3935 |
