# Supplementary material for: Reactive Transformation and Increased BDNF Signaling by Hippocampal Astrocytes in Response to MK-801
Source: PLoS One. 2015 Dec 23;10(12):e0145651. doi: 10.1371/journal.pone.0145651 (PMC4689377; doi:10.1371/journal.pone.0145651)
Supplement: S4 Table — (DOCX) [file pone.0145651.s013.docx]

**S4 Table. The data of BDNF protein by western blotting in vitro**

| BDNF | IOD | | |
| --- | --- | --- | --- |
|  | Ctrl | 5 uM | 20 uM |
|  | 1175 | 4065 | 3503 |
|  | 376 | 5614 | 5982 |
|  | 469 | 1411 | 1876 |
