# Supplementary material for: Reactive Transformation and Increased BDNF Signaling by Hippocampal Astrocytes in Response to MK-801
Source: PLoS One. 2015 Dec 23;10(12):e0145651. doi: 10.1371/journal.pone.0145651 (PMC4689377; doi:10.1371/journal.pone.0145651)
Supplement: S5 Table — (DOCX) [file pone.0145651.s014.docx]

**S5 Table. The data of TrkB protein by western blotting in vitro**

| TrkB | IOD | | |
| --- | --- | --- | --- |
|  | Ctrl | 5 uM | 20 uM |
|  | 1688 | 4694 | 6717 |
|  | 3280 | 10684 | 10848 |
|  | 1498 | 2382 | 2489 |
