# Supplementary material for: Reactive Transformation and Increased BDNF Signaling by Hippocampal Astrocytes in Response to MK-801
Source: PLoS One. 2015 Dec 23;10(12):e0145651. doi: 10.1371/journal.pone.0145651 (PMC4689377; doi:10.1371/journal.pone.0145651)
Supplement: S6 Table — (DOCX) [file pone.0145651.s015.docx]

**S6 Table. The data of p75 protein by western blotting in vitro**

| P75 | IOD | | |
| --- | --- | --- | --- |
|  | Ctrl | 5 uM | 20 uM |
|  | 635 | 1685 | 1106 |
|  | 576 | 3619 | 2417 |
|  | 713 | 2819 | 2908 |
